# 

**Published:** 1895-01

**Authors:** 


### CONTENTS.

ORIGINAL ARTICLES—

Tubercular Joint Disease in Children—A General Consideration. By Samuel E. Milliken, M. D., New York ........ 1
The Element of Causation in Abdominal Contusions. By Thos. H. Manley, M. D., New York ........ 4
Some Points in Rectal Surgery. By J. M. Mathews, M. D., Louisville, Ky ........ 9
Recto-Vaginal Fistulæ, and Fistulæ about the Anus in woman. By A. Lapthorn Smith, B. A., M. D., M. R. C. S., Eng., F. O. S., London, Montreal, Can ........ 16
Perforating Typhoid Ulcer—Peritonitis—Operation—Recovery. By Robert Abbe, M. D., New York ........ 22
A Postscript to the Report on Appendicitis. By W. S. Halsted, M. D., Baltimore, Md. ........ 28
Appendicitis. By Charles McBurney, M. D., New York ........ 32

SOCIETY REPORTS—

Maryland Clinical Society ........ 35

BOOK REVIEWS—

A Manual of Human Physiology. By Joseph H. Raymond, A. M., M. D .......... 41
Syllabus of Lectures on Human Embryology. By Walter Porter Manton, M. D .......... 41
Practical Analysis and Urinary Diagnosis. By Charles W. Purdy, M. D .......... 42
A Text-Book of the Diseases of Women. By Henry J. Garrigues, M. D .......... 43

SELECTIONS AND ABSTRACTS—

The Technique of Thiersch's Method of Skin-Grafting. By Theodore Dunham, M. D .......... 44

EDITORIALS—

Announcement .......... 48

Medical Examiners for the State of Georgia .......... 48

The Medical Law .......... 51

The Atlanta Society of Medicine .......... 55

Editorial Notes .......... 56

Prescriptions .......... 58

Special Notes .......... 60

